# *De Novo* Analysis of *Wolfiporia cocos* Transcriptome to Reveal the Differentially Expressed Carbohydrate-Active Enzymes (CAZymes) Genes During the Early Stage of Sclerotial Growth

**DOI:** 10.3389/fmicb.2016.00083

**Published:** 2016-02-03

**Authors:** Shaopeng Zhang, Bingxiong Hu, Wei Wei, Ying Xiong, Wenjun Zhu, Fang Peng, Yang Yu, Yonglian Zheng, Ping Chen

**Affiliations:** ^1^College of Biology and Pharmaceutical Engineering, Wuhan Polytechnic UniversityWuhan, China; ^2^Institute for Interdisciplinary Research, Jianghan UniversityWuhan, China; ^3^Hefei Enzyme Information Technology Co., LtdWuhan, China; ^4^College of Plant Protection, Southwest UniversityChongqing, China

**Keywords:** *Wolfiporia cocos*, sclerotial development, transcriptome, CAZymes, differentially expressed genes

## Abstract

The sclerotium of *Wolfiporia cocos* has been used as an edible mushroom and/or a traditional herbal medicine for centuries. *W. cocos* sclerotial formation is dependent on parasitism of the wood of *Pinus* species. Currently, the sclerotial development mechanisms of *W. cocos* remain largely unknown and the lack of pine resources limit the commercial production. The CAZymes (carbohydrate-active enzymes) play important roles in degradation of the plant cell wall to provide carbohydrates for fungal growth, development, and reproduction. In this study, the transcript profiles from *W. cocos* mycelium and 2-months-old sclerotium, the early stage of sclerotial growth, were specially analyzed using *de novo* sequencing technology. A total of 142,428,180 high-quality reads of mycelium and 70,594,319 high-quality reads of 2-months-old sclerotium were obtained. Additionally, differentially expressed genes from the *W. cocos* mycelium and 2-months-old sclerotium stages were analyzed, resulting in identification of 69 CAZymes genes which were significantly up-regulated during the early stage of sclerotial growth compared to that of in mycelium stage, and more than half of them belonged to glycosyl hydrolases (GHs) family, indicating the importance of *W. cocos* GHs family for degrading the pine woods. And qRT-PCR was further used to confirm the expression pattern of these up-regulated CAZymes genes. Our results will provide comprehensive CAZymes genes expression information during *W. cocos* sclerotial growth at the transcriptional level and will lay a foundation for functional genes studies in this fungus. In addition, our study will also facilitate the efficient use of limited pine resources, which is significant for promoting steady development of Chinese *W. cocos* industry.

## Introduction

*Wolfiporia cocos* (Schwein.) Ryvarden *et* Gilb. (Basidiomycota, Polyporaceae) is a fungus that parasitizes the roots of diverse species of *Pinus*. The fungus has a wide distribution in East Asia, including China, Japan, and Korea, and other regions of the world (Dai et al., 2009; Esteban, 2009; Wang et al., 2013). The sclerotium of *W*. *cocos*, also known as Fuling in China, has been used as an edible mushroom and/or a traditional herbal medicine for centuries (Dai et al., 2009; Kobira et al., 2012). Phytochemical and pharmacological research demonstrates that the major pharmacologically active components from Fuling, such as polysaccharides and triterpenes, possess a wide spectrum of pharmacological activities, including anti-viral., anti-tumor, anti-oxidant, free-radical scavenging, anti-rejection, anti-hyperglycemic, antibacterial., anti-inflammatory, and anti-hypertonic stress effects (Huang et al., 2007; Esteban, 2009; Rios, 2011; Feng et al., 2013; Wang et al., 2013; Zhao et al., 2013a; Tang et al., 2014).

*Wolfiporia cocos* sclerotial formation is dependent on parasitism of the wood of *Pinus* species (Kubo et al., 2006). However, commercial production of *W. cocos* sclerotia is currently limited by severe habitat destruction, ineffective protection, and the lack of *Pinus* species materials (Wang et al., 2012). Thus, exploration of novel artificial cultivation techniques that are independent of pine resources and the sustainable production of *W. cocos* sclerotia have attracted much interest. Studying the parasitic mechanisms of *W. cocos* on *Pinus* species and the genetic basis of sclerotial development will improve our understanding of the overall biology of the fungus and may facilitate commercial production.

With the development of next-generation sequencing technology, RNA deep-sequencing, especially the *de novo* sequencing, has been widely used for transcriptomic and functional genes study in several mushrooms (Liu et al., 2012; Yang et al., 2012; Shu et al., 2013). Nevertheless, compared with other fungi, the mechanisms of *W. cocos* sclerotial formation and biosynthesis of polysaccharides and triterpenes remain largely unknown, especially the associated transcriptomic data at early stage of sclerotial growth. Moreover, in other fungi, many studies have demonstrated that a series of important genes involved in sclerotial development are upregulated at early stage of sclerotial development (Yu et al., 2012a; Xing et al., 2013; Zhu et al., 2013; Xiao et al., 2014). Thus, sequencing and studying the *W. cocos* transcriptome of early stage of sclerotial growth will authentically provide great help for identifying more significant genes regulating sclerotial formation and other important economic traits.

To infect and colonize the plants tissues successfully, both saprophytic and phytopathogenic fungi secrete a diverse range of carbohydrate-active enzymes (CAZymes), especially cell wall degrading enzymes (CWDEs), to degrade barrier of the plant cell wall, which are known to be mainly composed of cellulose, hemicellulose, and pectin (Cosgrove, 2005). Based on catalytic activities associated motifs and/or domains, the CAZymes are functionally classified into six classes: glycosyl hydrolases (GHs), carbohydrate esterases (CEs), polysaccharide lyases (PLs), carbohydrate-binding modules (CBMs), glycosyltransferases (GTs), and auxiliary activities (AAs; Christian et al., 2014; Blackman et al., 2015).

The carbohydrates from degraded plant tissue, such as mono and oligosaccharides, can be utilized as nutrition for fungal growth, development, and reproduction. And a number of studies have also suggested that the nutrition conditions, such as optimal carbon source, play important roles on the sclerotial formation of different fungi (Rai and Agnihotri, 1971; Xing et al., 2011). Besides, when cultured on artificial medium, such as PDA, *W. cocos* could directly absorb and utilize small molecular nutrition source, such as glucose for mycelium growth, but it was unable to form sclerotia (Kubo et al., 2006). And the sclerotial formation and growth relied on colonization on the wood tissue of *Pinus* species to sustainably absorb and utilize the carbohydrates and other unknown nutrition source degraded from pine woods by the catalytic activities of CAZymes. In addition, several studies have also demonstrated that when fungi colonized on different host species or tissue components, hydrolytic enzymes showed activities preferences (King et al., 2011; Couturier et al., 2012). Thus, secretion of various CAZymes is a significant means by which *W. cocos* colonize and develop sclerotia successfully on the wood of *Pinus* species.

In this study, the transcript profiles from *W. cocos* mycelium and 2-months-old sclerotium, the early stage of sclerotial growth, were firstly analyzed using *de novo* sequencing technology, resulting in the identification of many differentially expressed genes (DEGs) during early stage of sclerotial formation, especially the CAZymes. And qRT-PCR was also used to confirm the expression profiles of these CAZymes genes. Our results will provide significant genes resource and facilitate future genes functional studies to gain a better understanding of sclerotial formation and growth mechanisms and other development processes of important economic traits. And data obtained in this study also provide a starting point to achieve a better understanding of the interaction system of *W. cocos*–*Pinus* species plants for efficient use of *Pinus* species wood and the steady development of Chinese *W. cocos* industry.

## Materials and Methods

### Strains and Culture Conditions

**FIGURE 1:**
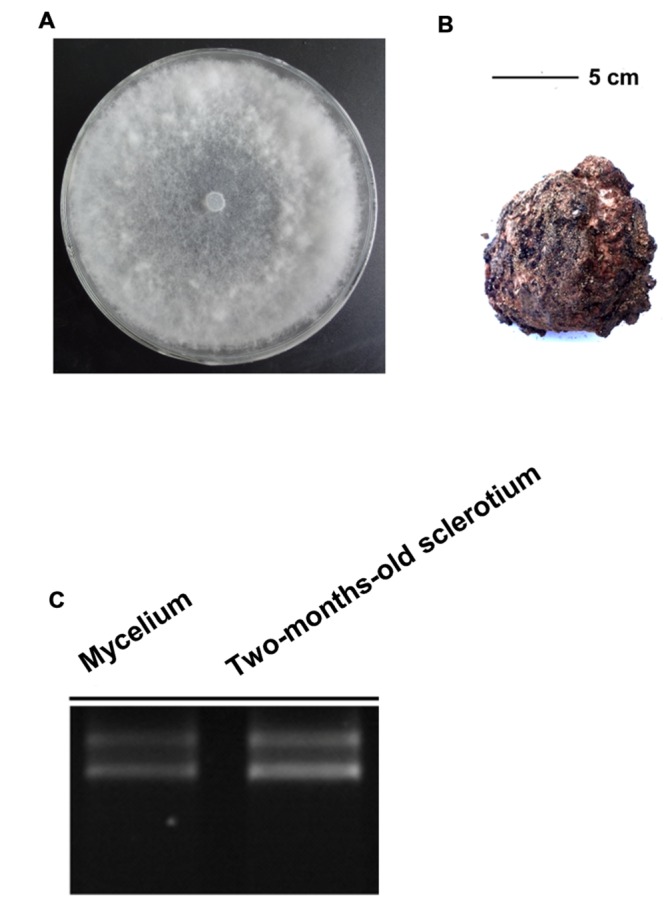
**The mycelium and sclerotia of *W. cocos* used for *De Novo* Sequencing. (A)** Colony morphology *W. cocos* mycelium grown on PDA for 7 days at 25°C. **(B)** *W. cocos* sclerotium of 2-months-old, scale bar = 5 cm. **(C)** The total RNA isolated from mycelium and 2-months-old sclerotium.

The *W. cocos* strain AS5.78 was obtained from the Agricultural Culture Collection of China, Institute of Soil and Fertilization, Chinese Academy of Agricultural Sciences, Beijing. *W. cocos* mycelia were grown on a cellophane membrane laid on potato dextrose agar (PDA) medium at 25°C for 7 days. Sclerotium of *W. cocos* strain AS5.78 at 2-months-old (**Figures 1A,B**) was obtained from the field in Luotian county, Hubei province, China. The mycelia and sclerotium of *W. cocos* were collected and immediately frozen in liquid nitrogen, then stored at –80°C for total RNA extraction.

### RNA Extraction, cDNA Library Construction, and Sequencing

Total RNA of mycelia and 2-months-old sclerotium were isolated with TriZOL reagent (Invitrogen, Carlsbad, CA, USA) according to manufacturer’s instructions (**Figure 1C**). The RNA quantity and quality were exampled with UV spectrophotometer (DU800, Beckman Coulter, USA) and gel electrophoresis, respectively, and then treated with DNase I (RNase Free; Takara, Dalian, China) to remove residual DNA according to manufacturer’s protocols. The construction of cDNA library was performed as described (Shu et al., 2013). Finally, the cDNA library was sequenced with Illumina HiSeq 2000 (Illumina Inc, San Diego CA, USA).

### Hybrid Assembly and Unigenes Function Annotation

We collected another RNA-seq data set from NCBI under accession number SRP018935 (Shu et al., 2013), which including two sample from mycelium and sclerotium. This dataset was included in all the following analysis. Before assembly, the raw reads contain adapters, and unknown (N) sequences were discarded. To obtain the high-quality clean reads, all reads were trimmed at the base with quality score lower than 20, and it will be discard if it is shorter than 50 bp. The individual reads will be kept if one of a pair reads is discarded. Transcript assembly was conducted in two steps. First, the reference genome of *W. cocos* was downloaded from JGI database^1^ and all clean reads were mapped on it using tophat2 (version 2.0.13) with default parameters, except that unmapped reads were output. Sequentially, cuﬄinks (v2.2.1) was used to assembly transcripts based on the map results. Second, all the unmapped reads from two datasets were combined and further assembly was conducted with trinity (version r20140413p1), and all the transcripts generated in this step were cluster with cd-hit-est (version 4.6), and both identity of 99% and coverage of 90% for shorter sequences were required and the longest sequence for each cluster were kept as the final unigenes together with transcripts generated by cuﬄinks.

Homologous sequences searching and functional annotation of all unigenes were performed by the using of BLASTx program against the NCBI NR database^2^, Swissprot^3^, and KEGG pathway^4^ (*E*-value <10^-5^).

### Expression Difference Analysis

As previously described (Yu et al., 2012b; Shu et al., 2013), the RPKM method (Reads per kb per Million reads) was used to calculate the unigene expression. The statistical method false discovery rate (FDR) was used in multiple tests to correct the threshold of *P*-value for the identification of DEGs between different developmental stages. In our analysis, we chose those with ratio ≥ 2 and FDR ≤ 0.001. The DEGs was analyzed with DEGSeq (Wang et al., 2010).

### CAZymes Annotation

The annotated unigenes from *W. cocos* transcriptome that are related to carbohydrate-active enzymes were further confirmed by the using of BLASTx program against CAZymes database^5^ at the threshold value of 1.0E^-20^. Unigenes possessing a sequence identity more than 60% with biochemically characterized CAZymes were considered as candidate.

### qRT-PCR Confirmation of CAZymes Genes

Total RNA of mycelia and sclerotia were extracted and treated with DNase I as described above. And the RevertAid^TM^ First Strand cDNA Synthesis Kit (MBI Fermentas, Lithuania) was used to generate the first strand cDNA according to the manufacturer’s protocols. Gene expression was analyzed by qRT-PCR using a Bio-Rad CFX96 Real Time System (Bio-Rad, USA) and QuantiTect SYBR Green PCR master mix (Bio-Rad, USA), according to the manufacturer’s instructions. The PCR conditions were as follows: denaturation at 94°C for 3 min; 40 rounds of 94°C for 20 s, 55°C for 20 s, and 72°C for 30 s; final step of 72°C for 10 min. The primers for qRT-PCR are listed in **Supplementary Table S1**. The expression of *W. cocos alpha-tubulin* gene (F:5′ACTCCAGCTTGGACTTCTTG3′ and R: 5′TCTTCGTCTTCCACTCCTTTG3′; Shu et al., 2013) was used to normalize the RNA sample for each qRT-PCR. For each gene, qRT-PCR assays were repeated at least three times, with each repetition having three technical replicates.

## Results

### Illumina Sequencing and *De Novo* Assembly

To obtain an overview of the *W. cocos* genes expression profiles during early growth stages, cDNA samples from mycelium and 2-months-old sclerotium were sequenced by using an Illumina HiSeq2000 platform, and the comparative analysis between mycelium and 2-months-old sclerotium was studied. After filtering the adapters, low quality and unknown (N) sequences, a total of 142,428,180 high-quality reads of mycelium and 70,594,319 high-quality reads of 2-months-old sclerotium were obtained.

**FIGURE 2:**
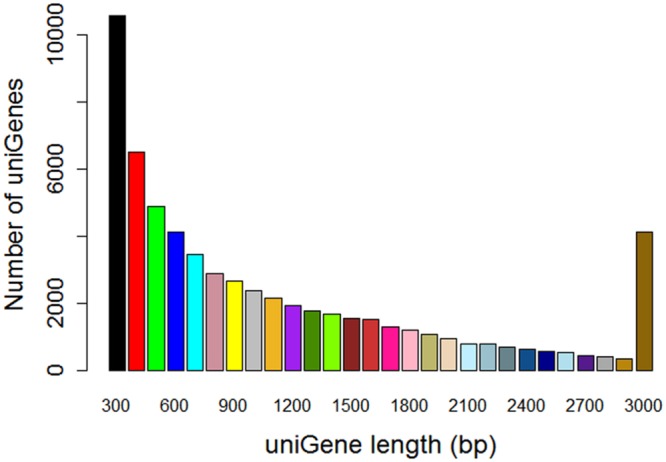
**Assembled unigenes length distribution of *W. cocos* transcriptome from mycelium and 2-months-old sclerotium**.

**FIGURE 3:**
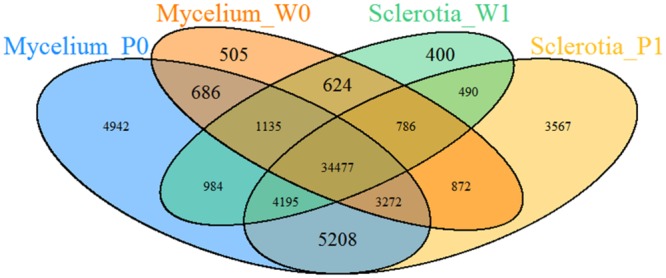
**The number of unigenes for mycelium and sclerotium from different two data sets.** Mycelium_P0 and Sclerotia_P1 were from the data set sequence in this project and Mycelium_W0, Sclerotia_W1 were from Shu et al. (2013).

Then, by the using of Cuﬄinks and Trinity software, these high-quality reads were assembled together to totally generate 62,143 unigenes with length above 200 bp. For the all-unigenes set, the average length of the was 1114 bp, the max and min length were 16,339 bp and 201 bp, respectively. About 43.54% (27,059) of all-unigenes were longer than 1,000 bp (**Figure 2**). In summary, 57,686 unigenes from mycelium, 56,010 unigenes from 2-months-old sclerotium in our data set, and 42,357 unigenes from mycelium, 43,091 unigenes from matured sclerotium in data set of Shu et al. (2013) were obtained in (**Figure 3**), respectively.

### Functional Annotation of *W. cocos* Transcriptome

BLASTX alignments (*E*-value cut-off of 10^-5^) of all unigenes sequences against several protein databases, including NBCI non-redundant (NR), Swiss-Prot, GO, and KEGG, showed that, respectively 10,137, 4,983, 8,024, and 7,152 unigenes could match significantly to proteins with known biological functions (**Table 1**), whereas the remainder are currently lacking in the database. Most of them were short fragments, and some of them might be non-coding RNA sequences or new genes.

**Table 1:** All-in-one list of annotations.

Annotation database	No. of annotation	Percent of annotation (%)
Total Unigene	62,143	100%
NR	10,137	16.31%
Swiss-Prot	4,983	8.02%
GO	8,024	12.91%
KEGG	7,152	11.51%


**FIGURE 4:**
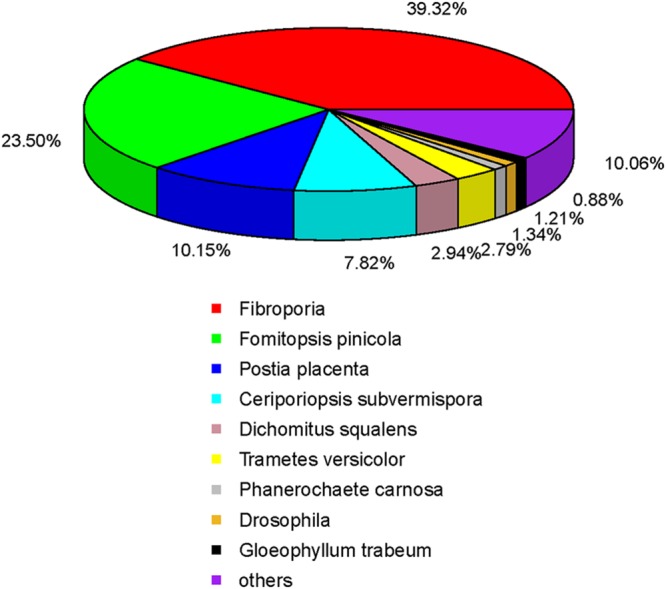
**Distribution of the homology search of expressed sequence tags against the nr database**.

The species distribution of the top BLASTX match against the NR database for the *W. cocos* unigenes showed that about 39.32% of *W. cocos* unigenes match with genes of *Fibroporia*, followed by *Fomitopsis pinicola* (23.50%), *Postia placenta* (10.15%), *Ceriporiopsis subvermispora* (7.82%), *Dichomitus squalens* (2.94%), *Trametes versicolor* (2.79%), and *Phanerochaete carnosa* (1.34%). The rest of 12.15% *W. cocos* unigenes had first hits with other fungal species (**Figure 4**).

### Analysis of Differentially Expressed Genes During Sclerotial Formation

**FIGURE 5:**
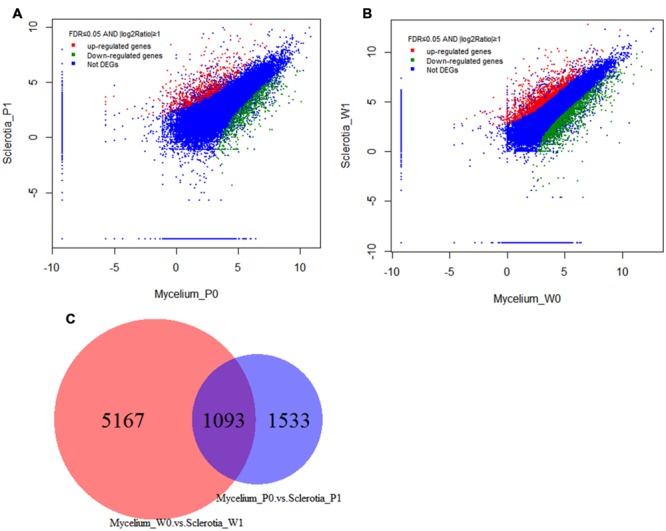
**Gene transcription profile between mycelium and sclerotial stages.** Scatter plot of total unigenes from the *W. cocos* transcriptome. The data was normalized as RPKM values and represented on a log_10_ scale. Red areas represent up-regulated unigenes in sclerotia, green areas represent down-regulated unigenes in the sclerotia and the blue areas represent no significant expression difference unigenes between mycelium and sclerotia. **(A)** Sclerotia_P1 and Mycelium_P0 were from the data set sequence in this project. **(B)** Sclerotia_W1 and Mycelium_W0 were from Shu et al. (2013). **(C)** Differentially expressed unigenes in both data sets.

For identification of DEGs between *W. cocos* mycelium and early sclerotium growth stages, the expression data of unigenes were analyzed with DEGSeq. Compared with mycelium, 1,371 and 1,255 unigenes showed upregulated and downregulated, respectively, in 2-months-old sclerotium (**Figure 5A**), 3,052 and 3,208 unigenes showed upregulated and downregulated, respectively, in matured sclerotium were identified in the data set of Shu et al. (2013), (**Figure 5B**). One thousand and ninety-three unigenes were differentially expressed in both data sets (**Figure 5C**).

### CAZymes Genes In *W. cocos*

Carbohydrate-active enzymes, including Glycoside hydrolases (GHs), CEs, AAs, CBMs, GTs, and PLs, are involved in carbohydrate metabolism. In our study, the BLASTx results indicated that a total of 306 unigenes from *W. cocos* transcriptome was identified to CAZymes. Among of these unigenes, 181 homologs belonged to the GHs, 88 candidates to GTs, 28 candidates to CEs, four candidates to CBMs, three to AAs, and two to PLs (**Supplementary Table S2**). In the GHs, GH16 and GH5 family both contained 28 unigenes, followed by GH18 (16 unigenes), GH28 (13 unigenes), GH31 (11 unigenes), GH3 and GH13 (both nine unigenes). In the GTs class, GT8 and GT2 both possessed 12 unigenes and was the most dominant, followed by GT4 (seven unigenes), GT20 (six unigenes), and GT15 (five unigenes). In CEs, CE4, and CE16 both contained seven unigenes, followed by CE10 (six unigenes). In CBMs, three unigenes was CMB21 and one was CMB43. There were only two PL14 in PLs class and three AA6 in AAs class (**Supplementary Table S2**).

**FIGURE 6:**
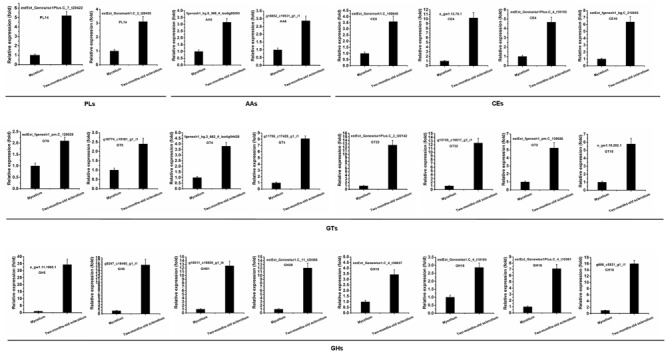
**qRT-PCR validation of differentially expressed CAZymes genes.** The relative expression of target genes in mycelium stage was set as level 1. Expression of *W. cocos alpha-tubulin* gene was used to normalize different samples. Bars represent means and standard deviations (three replications).

The differential expression of these CAZymes genes was evaluated. The results indicated that 69 CAZymes genes were significantly up-regulated (≥1.5-fold) in the 2-months-old sclerotium compared to that of in mycelium (**Supplementary Table S2**, yellow fluorescence label), containing 43 GHs, 17 GTs, five CEs, two PLs, and two AAs. Then, some of these up-regulated genes were selected to further examined the expression pattern by the using of qRT-PCR (**Figure 6**) and the results correspond with *de novo* transcriptome sequencing data. The results suggested that these up-regulated CAZymes unigenes is involved in *W. cocos* sclerotial formation possibly when colonize on pine wood at the early stage.

## Discussion

Sclerotia of *W. cocos* possess important medicinal value and have been widely used in East Asia for centuries (Dai et al., 2009; Kobira et al., 2012). The sclerotial formation is dependent on parasitism of the wood of *Pinus* species (Kubo et al., 2006). At present, the lack of *Pinus* species materials currently limits the commercial production of *W. cocos* sclerotia. Thus, the efficient use of limited pine resources is significant for promoting steady development of Chinese *W. cocos* industry.

While the pharmacologically activities of polysaccharides and triterpenes have been extensively studied (Esteban, 2009; Wang et al., 2015a,b), the genomic and transcriptomic information remain largely unknown on this fungus, especially for the CAZymes genes which play important roles in carbohydrate degradation and plant tissue colonization (Kubicek et al., 2014). In this study, the transcriptome of *W. cocos* from mycelium and 2-months-old sclerotium were analyzed and numerous DEGs, especially the CAZymes genes relating to carbohydrates degradation, were identified. Comparative analysis with the data of Shu et al. (2013) revealed that *W. cocos* highly expressed CAZymes genes, especially in the early stage of sclerotial development, which supports with the facts that *W. cocos* needs to sustainably absorb and utilize the carbohydrates and other unknown nutrition source degraded from pine woods for sclerotial growth.

Among these CAZymes genes, the GHs family was the most dominant prevalent. These results are similar to those previously described by Yu et al. (2012b) in *Ganoderma lucidum* (Basidiomycota, Polyporaceae). GHs mainly hydrolyze the glycoside bond between two or more carbohydrates or between a carbohydrate and a non-carbohydrate (Cantarel et al., 2009). To date, based on amino acid sequence, GHs are classified into 127 families and 91 of them were found in fungi (Zhao et al., 2013b). According to our transcriptome data, families GH16, GH18, GH28, and GH5 were the dominant among these up-regulated CAZymes genes in the early stage of sclerotial development (**Supplementary Table S2**). GH16 family enzymes possess xyloglucanase activity and may degrade β-1,3-glucans or xyloglucans, which play significant roles in hemicellulose degrading (Baumann et al., 2007; Nakajima et al., 2012). GH18 may degrade chitin and cleave the β-1,4-linkage between *N*-acetylglucosamine residues at the base of the oligosaccharide chain while GH28 possess polygalacturonases activity and play a critical role in pectin degradation (Zhao et al., 2013b; Blackman et al., 2015). GH5 is one of the largest GH families and consists of a wide range of enzymes activity on different substrates, such as β-1,3-glucans, β-1,4-glucans in cellulose, and β-1,4-mannans in hemicellulose (Aspeborg et al., 2012; Blackman et al., 2014). In our study, six GH5, 10 GH16, five GH18, and six GH28 genes showed up-regulated expression in the early stage of sclerotial growth (2 months; **Supplementary Table S2**), suggesting that these enzymes play important roles in plant cell wall degradation to provide sufficient nutrition for the growth of *W. cocos* sclerotium.

In addition, as *W. cocos* is unable to form sclerotium in the absence of pine woods, thus we inferred that some special components from *Pinus* species plants could induce the formation and development of *W. cocos* sclerotium, and these unknown components will also definitely induce the differential expression of genes involved in *W. cocos* sclerotial development. Characterization of these potential candidates of sclerotial development associated genes will be helpful to elucidate the mechanism of sclerotial formation. And studying on the DEGs involved in chemical components synthesis and metabolism will also give a new clue to identify the components involved in inducing *W. cocos* sclerotial formation.

In summary, we have analyzed the *W. cocos* transcriptome in mycelium and early stages of sclerotial growth, resulting in identifying various DEGs, especially the CAZymes genes. Characterization of these genes will enhance our understanding on mechanism of *W. cocos* sclerotial development. However, our finding also arise more questions to be answered, such as, what kinds of components promote *W. cocos* sclerotial formation? Do these components only come from pine woods or from the interaction product of *W. cocos*-host? And how do these components induce *W. cocos* sclerotial formation?

## Conclusion

This study contributes to a better understanding of the DEGs during the early stage of *W. cocos* sclerotial growth in comparison with that of mycelium stage, especially the CAZymes genes. And it also provides a valuable first step toward the understanding and analysis of the mechanism of *W. cocos* sclerotial development and interaction system of *W. cocos*-pine woods.

## Author Contributions

Conceived and designed the experiments: WZ. Performed the experiments: SZ, BH, WW, YX, and WZ. Analyzed the transcription data: SZ, BH, WW, YX, WZ, FP, and YY. Contributed reagents/materials/analysis tools: WW, WZ, PC, and YZ. Wrote the paper: WW, WZ, PC, and YZ. All authors read and approved the final manuscript.

## Conflict of Interest Statement

The authors declare that the research was conducted in the absence of any commercial or financial relationships that could be construed as a potential conflict of interest.

## Footnotes

1 http://genome.jgi.doe.gov/Wolco1/Wolco1.home.html

2 http://www.ncbi.nlm.nih.gov/

3 http://www.expasy.ch/sprot/

4 http://www.genome.jp/kegg/

5 http://www.cazy.org/

## Supplementary Material

TABLE S1 **Primers for qRT-PCR to verify *W. cocos* CAZymes genes**.

TABLE S2 **Differentially expressed carbohydrate-active enzymes (CAZymes) genes between *W. cocos* mycelium and 2-months-old sclerotium.** The up-regulated genes were labeled with yellow fluorescence label.
