# Supplementary material for: De Novo Analysis of Wolfiporia cocos Transcriptome to Reveal the Differentially Expressed Carbohydrate-Active Enzymes (CAZymes) Genes During the Early Stage of Sclerotial Growth
Source: Front Microbiol. 2016 Feb 3;7:83. doi: 10.3389/fmicb.2016.00083 (PMC4738778; doi:10.3389/fmicb.2016.00083)
Supplement: TABLE S1 — Primers for qRT-PCR to verify W. cocos CAZymes genes. [file Table_1.DOCX]

**TABLE S1 Primers for qRT-PCR to verify *W. cocos* CAZymes genes**

| **Unigene_ID** | **CAZY family** | **Primer** | **sequence(5’ to 3’)** |
| --- | --- | --- | --- |
| fgenesh1_kg.9_309_#_isotig05093 | AA6 | 1-F | ATGTGTATCCCTGGTAAACGCA |
|  |  | 1-R | CGACGCCGACCTTGACGGACTC |
| g18852_c19531_g1_i1 | AA6 | 2-F | GTTGCTATCGTCTTCTATTCCC |
|  |  | 2-R | GGGATACCGAAGAGGAAAGCAT |
| estExt_fgenesh1_kg.C_210055 | CE10 | 3-F | ATGATGCTGTCCTCTCTTCT |
|  |  | 3-R | AGCGAATGGCATCCCTAAGAAG |
| e_gw1.13.70.1 | CE4 | 4-F | CACGGTGACTGTTGGGAGCGAA |
|  |  | 4-R | CTTGAGCACATTCGCCCTTTCC |
| estExt_Genewise1Plus.C_4_t10105 | CE4 | 5-F | AGAGACGCTGGGCACGAGATAG |
|  |  | 5-R | TCTGTGCCTTCTTTGCTTGTCT |
| estExt_Genemark1.C_100045 | CE8 | 6-F | CTGGATTGTTGTTTGCCATTTC |
|  |  | 6-R | CGGAAAGGAACCAGAAAGTCGT |
| estExt_Genewise1Plus.C_4_t10361 | GH16 | 7-F | ATGTCCCTCCCCGACCTCCGCC |
|  |  | 7-R | GGTAGGGCGAGTCGACGGCAG |
| g686_c5831_g1_i1 | GH16 | 8-F | AAGCGACTCTTCTTCAGTGTTC |
|  |  | 8-R | CGGTTGCTCTTGCGTCCAGGGC |
| estExt_Genewise1.C_4_t10184 | GH18 | 9-F | ATCAATGCGGTCAATACTTACA |
|  |  | 9-R | CAGTGAAAAATGAGAGGAGGTT |
| estExt_Genewise1.C_4_t30037 | GH18 | 10-F | AGTCTGTATGCTGGGTGGAGTG |
|  |  | 10-R | GTCAGATCCCACTGGTCCGTGT |
| estExt_Genewise1.C_11_t20365 | GH28 | 11-F | AGGCTTGGTTGCCACACTCTCG |
|  |  | 11-R | ATACCAAACCTGACCATTCCCG |
| e_gw1.11.1065.1 | GH5 | 12-F | ATGCGTAGAATATCACAAT |
|  |  | 12-R | ATCGCTCAAGGACAAACCAG |
| g9247_c16460_g1_i1 | GH5 | 13-F | CATGTCCGAGTAAGGCTAGC |
|  |  | 13-R | AATCGTCAAGTCGTGTCAGTC |
| g16811_c18808_g1_i4 | GH51 | 14-F | GCAACGCAGGATACGGAGGAAT |
|  |  | 14-R | TCGGTCTGTTCTTGTATGTCGG |
| e_gw1.10.202.1 | GT15 | 15-F | AGTTCAATAAGCAGTTTGGGTA |
|  |  | 15-R | GATAAGAACCGATGGCATACCT |
| estExt_fgenesh1_pm.C_130026 | GT2 | 16-F | GTGCCGTTTGGTGTCATAGG |
|  |  | 16-R | GTAGGGGACGGTCAACACGC |
| estExt_fgenesh1_pm.C_120029 | GT8 | 17-F | TGAACATGGTCAACACGACG |
|  |  | 17-R | CTTCGTGTACTGGTCGACGAAG |
| g18774_c19161_g1_i1 | GT8 | 18-F | GCTACGATGACGTAACGGTC |
|  |  | 18-R | TGCATGACGATGTACGTACGT |
| estExt_Genewise1Plus.C_2_t20142 | GT22 | 19-F | TCGCGTGGACCGTCGGCGTGT |
|  |  | 19-R | TCGCGTCCACGAGCGACTT |
| g10158_c16817_g1_i1 | GT32 | 20-F | GACGCAAATGCCTACATCACCG |
|  |  | 20-R | GTGGTAGAGAATGAAGTAGCGG |
| fgenesh1_kg.2_662_#_isotig04428 | GT4 | 21-F | TGGGATTCGCAGTTCTACAT |
|  |  | 21-R | TTACGACGATGTCTGAGGCA |
| g11706_c17428_g1_i1 | GT4 | 22-F | CGGGAGCGGATACGCCTACAGT |
|  |  | 22-R | AAACTCTTCCAGGTCGTCAATC |
| estExt_Genemark1.C_2_t20450 | PL14 | 23-F | CCCTTTCGGTGATGTCTGATGTT |
|  |  | 23-R | CGAGTTTGCCGGATAAAACAG |
| estExt_Genewise1Plus.C_7_t20422 | PL14 | 24-F | TCTGTCACCAGTATTGCCCT |
|  |  | 24-R | CGAGATTATTTCCGCCGTCC |
